# Evaluation of a Telehealth Counseling Program for Expatriates

**DOI:** 10.1089/tmj.2018.0082

**Published:** 2019-08-01

**Authors:** Hwa Yeon Park, Ju Young Kim, Hye Yeon Koo, Jinah Han, Ji Hye Jun, Wonjae Lee, Ki Young Na, Hyang Yuol Lee, Yuliya Pak, Seunghee Jang, Sergey Kim, Chulkyu Jeong, Taewook Nam

**Affiliations:** ^1^ Health Promotion Center, Gachon University Gil Medical Center, Incheon, Korea.; ^2^ Department of Family Medicine, Seoul National University Bundang Hospital, Seongnam, Korea.; ^3^ Office of External Affairs, Seoul National University Bundang Hospital, Seongnam, Korea.; ^4^ Department of Family Medicine, Chamjoeun Hospital, Gwangju, Korea.; ^5^ Department of Cardiology, Seoul National University Bundang Hospital, Seongnam, Korea.; ^6^ International Healthcare Center, Seoul National University Bundang Hospital, Seongnam, Korea.; ^7^ Research Institute of Healthcare Policy, Seoul National University Bundang Hospital, Seongnam, Korea.; ^8^ Department of Nephrology, Seoul National University Bundang Hospital, Seongnam, Korea.; ^9^ Ushin C & C, Ansan, Korea.

**Keywords:** *expatriates*, *occupational travelers*, *telehealth counseling*, *feasibility*, *acceptability*, *telemedicine*

## Abstract

***Background:*** *Health problems for expatriates are common due to their vulnerability to local infectious diseases, psychosocial problems, and chronic diseases, but many problems go largely unmet in this unique population.*

***Introduction:*** *Telehealth counseling was developed and tested for Korean expatriates. We explored the current status of using telehealth counseling systems and showed its feasibility and acceptability in three countries.*

***Materials and Methods:*** *This retrospective study was based on the “Development and demonstration of telehealth counseling program for overseas Koreans” project funded by the Korea Health Industry Development Institute. In this project, we established five Digital Healthcare Centers (DHCs): 3 in Vietnam and 1 each in Uzbekistan and Cambodia. We used data from October 2016 to September 2017; descriptive analysis and one-way ANOVA were used to present detailed information.*

***Results:*** *A total of 442 patients made an appointment for telehealth counseling services. Overall user satisfaction rates were 96.1%. Over two thirds of patients (302/442, 68.3%) completed one-time telehealth counseling. About 13% were referred to primary care, and 17 (3.8%) were referred to specialists or tertiary hospital. The most common diagnostic category was endocrine, nutritional, and metabolic diseases (14%), followed by diseases of the circulatory system (12.3%) for one-time visit patients.*

***Discussion:*** *Our telehealth counseling program for expatriates was feasible and acceptable in three countries. It also has the potential to minimize language barriers and the cost of healthcare usage.*

***Conclusion:*** *Further research for sustainable effective telehealth systems for expatriates will be needed.*

## Introduction

Every day, over 3 million international travelers cross borders^1^ and some 15% of them travel for business and professional purposes.^2^ Rapid globalization makes migration and expatriation grow substantially. In Korea, around 7 million Koreans (15% of all Koreans, including those who have immigrated to other countries) live abroad, mostly in China or the United States, but recently, Korean expatriates to southeast Asian countries such as Vietnam have been rapidly increasing.^3^ In general, an expatriate is defined as an individual who moves to another country, changes their dominant place of residence, and obtains legal work abroad.^4^

Among expatriates, 25–36% reported health problems.^5,6^ Age, destination country, and climate or cultural difference from the original country were factors associated with health problems.^6^ Risks and diseases associated with both common and uncommon destinations could be more frequently experienced by the expatriates. Behavioral and psychosocial problems were also quite common, and adjustment disorders or depression was more frequently found among expatriates.^5,6^ Moreover, they could experience occupational health risks, such as chemical, physical, ergonomic, and biological hazards, and location-specific exposure to transmissible diseases, such as viral hepatitis, malaria, or enteric infections.

Recently, telemedicine has emerged as a paradigm-shifting strategy, especially in developing countries, using “initiate-build-operate-transfer (IBOT)” methods.^7^ IBOT includes assessment of healthcare needs, the establishment of a telemedicine center and network, the empowerment of telemedicine experts, and the integration of the telemedicine program into the local public institution. Moreover, telemedicine technologies have been transformed to be cheaper and faster, while breaking barriers of time and distance.^8^ These telemedicine systems can be optimized to manage health problems for expatriates who are a vulnerable segment of the population. Despite its potential to promote quality of life and accessibility to healthcare among expatriates, telehealth studies on this population have never, as far as we know, been carried out.

In Korea, the government funded the pilot project for the telehealth counseling program. As part of the project, we provided telehealth counseling programs for Korean expatriates in Vietnam, Cambodia, and Uzbekistan.

This study aimed to describe our implementation of the telehealth counseling system and demonstrate the utilization pattern, feasibility, and acceptability of telehealth counseling programs for Korean expatriates.

## Materials and Methods

### Establishing Digital Healthcare Center

#### Establishment

This study was based on the “Development and demonstration of telehealth counseling program for overseas Koreans” project funded by the Korea Health Industry Development Institute.

We established global Digital Healthcare Centers (DHCs) for overseas Korean populations in Vietnam, Uzbekistan, and Cambodia as a government-funded project; these were connected with Seoul National University Bundang Hospital. These sites were chosen based on the location of Korean Association centers (because their help with support was needed in establishing DHCs) and also in relation to the location of collaborating hospitals, just in case further referral for medical diagnosis with treatments became necessary during the teleconsultation.

In Vietnam, a Korean Association was not easily available for helping with the establishment of a DHC; hence, we established one in the Korean Vietnamese News Agency and two in local hospitals or international clinics. In Cambodia and Uzbekistan, we established DHCs within the Korean Association center with permission.

Participants were usually recruited by announcements through overseas Korean Associations; a Korea–Vietnam news organization in Ho Chi Minh City also helped recruit participants through newspaper advertisements for free. We also received help from the consulates and ambassadors in notifying and promoting our DHCs to overseas Koreans.

Coordinators were recruited through local employment portals or flyers from overseas Korean Associations. Coordinators were required to have Korean nursing certificates to be in charge of each center. The coordinator at each center provided patients with information about the services and received their informed consents. They were responsible for patient registration, making appointments, anthropometric measurements, checking vital signs, and patient education. They were also allowed to triage participants, and, if patients needed urgent or emergency medical service, patients were recommended to go to a hospital or emergency room with a doctor's approval.

In 2016, a total of 6 family physicians were involved. In 2017, a total of 19 doctors with different specialties such as urologist, obstetrician & gynecologist, pediatrician, neurologist, neurosurgeon, ophthalmologist, otolaryngologist, psychiatrist, orthopedic surgeon, and dermatologist participated in the telehealth counseling program.

#### Software architecture

As a part of organizing the DHCs, internet connections were installed, and video consultation equipment was set up for face-to-face counseling in each country. We built a telehealth counseling system with a tele-video conference system by AVAYA^®^ and web browsers connected with each DHC. Each DHC had an anthropometric measurement device, glucometer, blood pressure monitoring device, and thermometer.

Our system permitted point-to-point connection only, so a central site could be involved in the telehealth counseling with each DHC at a given time. Thus, if the main center had to serve requests from five remote DHCs, the telemedicine center would have to serially respond to one request at a time.

We offered synchronous connection with patients in each DHC, including transfer of information if needed. Information included radiologic images, dermatologic pictures, or consultation notes from local hospitals. Coordinators preregistered such information on their website, so that during the telehealth counseling, doctors could easily access the information, and provided second opinions as well.

Our telehealth system is a fixed terminal such that patients could come to DHC for telehealth counseling that is connected with a domestic hospital. This system is also connected through a virtual private network (VPN) so that patient information can be stored safely and securely. The server was located in a hospital network system to prevent damage to the physical environment from factors such as temperature, humidity, or dust and to securely store it.

#### Counseling system flow

People booked an appointment for counseling and filled out the initial questionnaire by visiting or calling the center. The coordinator then checked the content to see if they needed hospital care immediately or if the images of previous medical reports they brought had to be read before counseling. Scheduled counseling was performed daily for an hour. When a patient visited the DHC on a reserved date, coordinators measured a patient's height, weight, blood pressure, and body temperature and entered the records in the web page.

**Fig. 1.**
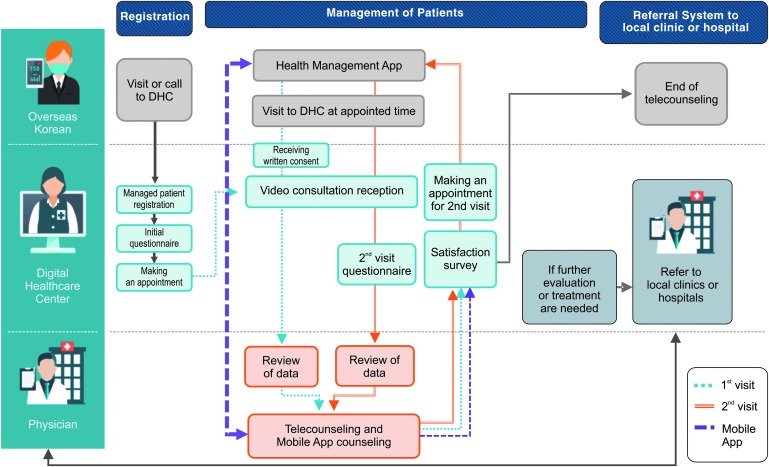
Counseling system flow. DHC, digital healthcare centers. Color images are available online.

In the telehealth counseling system, doctors in South Korea asked patients questions and performed a proxy examination with the assistance of the nurses in the remote countries. In the case of a typical one-time visit, no follow-up session was included. If patients had hypertension or diabetes, we provided them with chronic disease management programs, including equipment for measuring blood pressure or glucose, and educational material with mobile apps for remote monitoring in Vietnam.^9^ In case of a need for further evaluation, patients were referred to local medical institutions. After finishing the telehealth counseling, patients were required to indicate user satisfaction through self-administered questionnaires (*Fig. 1*).

### Study Design and Participants

This was a retrospective study using the data from five DHCs between October 1, 2016 and September 30, 2017. Study participants were Korean expatriates who had lived in the corresponding country for a minimum of over 3 months and had not lost their Korean nationality. A total of 442 patients in the three countries visited the centers to take part in the telehealth counseling program.

### Evaluation Tool

Complaints from participants were recorded based on the International Classification of Diseases 10th Revision (ICD-10). User satisfaction was evaluated through self-administered questionnaires after finishing the telehealth counseling, regarding the doctor, coordinator, accessibility, booking appointments, and in general, using 100 visual analog scales.

Baseline demographic information, anthropometric measurements, past medical histories, and current medication use were obtained through the coordinator.

### Ethical Considerations

This study was approved by the Institutional Review Board in Seoul National University Bundang Hospital (No. B-1708/412-130). All research activities in this study were conducted in accordance with the ethical principles of the Declaration of Helsinki.

### Statistical Analysis

We used the STATA 14.0 program to perform statistical analysis. The patient characteristics were described with means and standard deviation in continuous variables or frequencies with percentages in categorical variables. We compared satisfaction with the telehealth counseling program with a one-way ANOVA among the three countries. Commonly encountered complaints were described with percentages in ICD-10 codes according to one-time users versus frequent users (more than three visits). For the referral cases, commonly encountered complaints were also described with percentages in ICD-10 codes according to local clinic versus tertiary hospital.

## Results

A total of 442 patients visited DHCs for telehealth counseling services. Among them, more than half were in their forties (54.8%) and lived in Vietnam (65.4%) (*Table 1*).

**Table 1.** Baseline Characteristics of Participants Visiting Digital Healthcare Center (*N* = 442)

	*N*	%
Gender		
Men	202	45.7
Women	240	54.3
Age group, years		
1–19	83	18.8
20–39	41	9.3
40–59	243	55.0
≥60	75	17.0
Country		
Vietnam	289	65.4
Uzbekistan	105	23.8
Cambodia	48	10.9
Transportation to DHC		
Walking	130	30.9
Car	139	33.0
Taxi	114	27.1
Public transportation	8	1.1
Motorcycle	30	7.1
Access time, min		
<30	342	81.0
30–59	56	13.3
≥60	24	5.7
Receiving chronic disease management program		
With only HT	57	12.9
With only DM	29	6.6
Both HT and DM	18	4.1

DHC, digital healthcare center; DM, diabetes mellitus; HT, hypertension.

The overall patient satisfaction rate was 96.1%. There was a difference in satisfaction rate among the three countries (*p* = 0.002). The average counseling time was 11 ± 0.27 min with differences among three countries, and most counseling sessions were conducted in Vietnam (*Table 2*). Nearly two thirds of participants (302/442, 68.3%) received one-time telehealth counseling, and 79 (17.9%) had a follow-up session; 80 (18.1%) were referred to primary care, and 17 (3.8%) were referred to specialists or to tertiary hospitals (*Table 3*).

**Table 2.** Satisfaction Rates of Telehealth Counseling Program According to Three Countries

	TOTAL	VIETNAM	UZBEKISTAN	CAMBODIA	*p* VALUE
Number of consultations, *N* (%)	442 (100)	289 (65.4)	105 (23.8)	48 (10.9)	
Satisfaction rate,%	96.1	96.5	95.7	94.6	0.002
Counseling duration (min)	11 ± 0.27	11.67 ± 0.39	10.39 ± 0.37	9.1 ± 0.67	<0.001

**Table 3.** Characteristics of Consultations in Digital Healthcare Center (*N* = 442)

	*N*	%
Patients having consultations		
Once	302	68.3
Twice	79	17.9
Thrice or more	27	6.1
Referral case		
To local clinics^a^	80	18.1
To hospitals^b^	17	3.8

^a^ In need of primary care.

^b^ In need of specialists or needed to go to tertiary hospital.

**Fig. 2.**
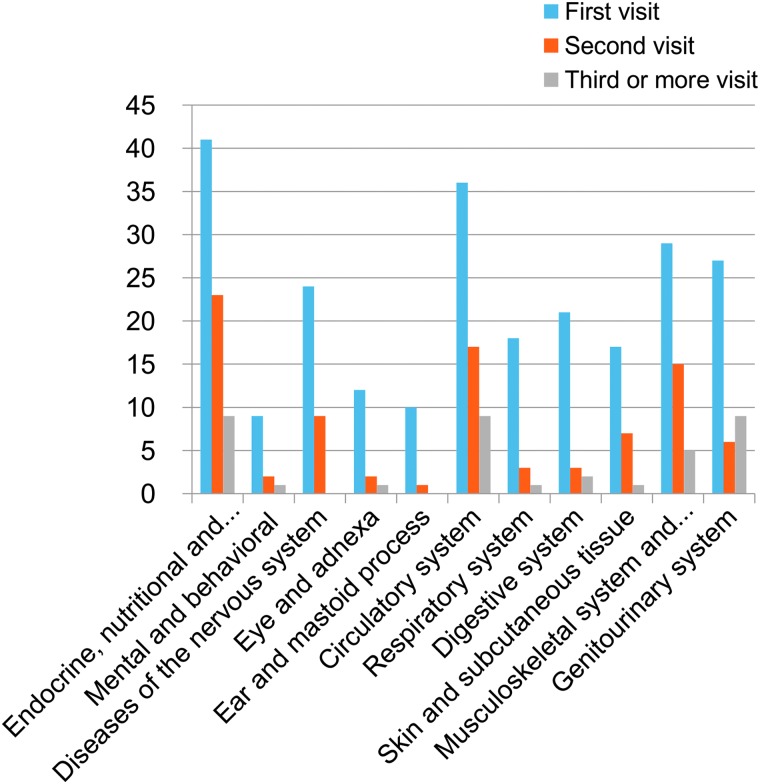
Classification of diagnosis according to the number of visits. Color images are available online.

The most common diagnostic category was endocrine, nutritional, and metabolic diseases, followed by diseases of the circulatory system and the nervous system for one-time visit patients. For patients who visited more than twice, diseases of the genitourinary system; endocrine, nutritional, and metabolic diseases; and diseases of the circulatory system had the same proportion of diagnostic categories, followed by diseases of the musculoskeletal system (*Fig. 2*).

**Fig. 3.**
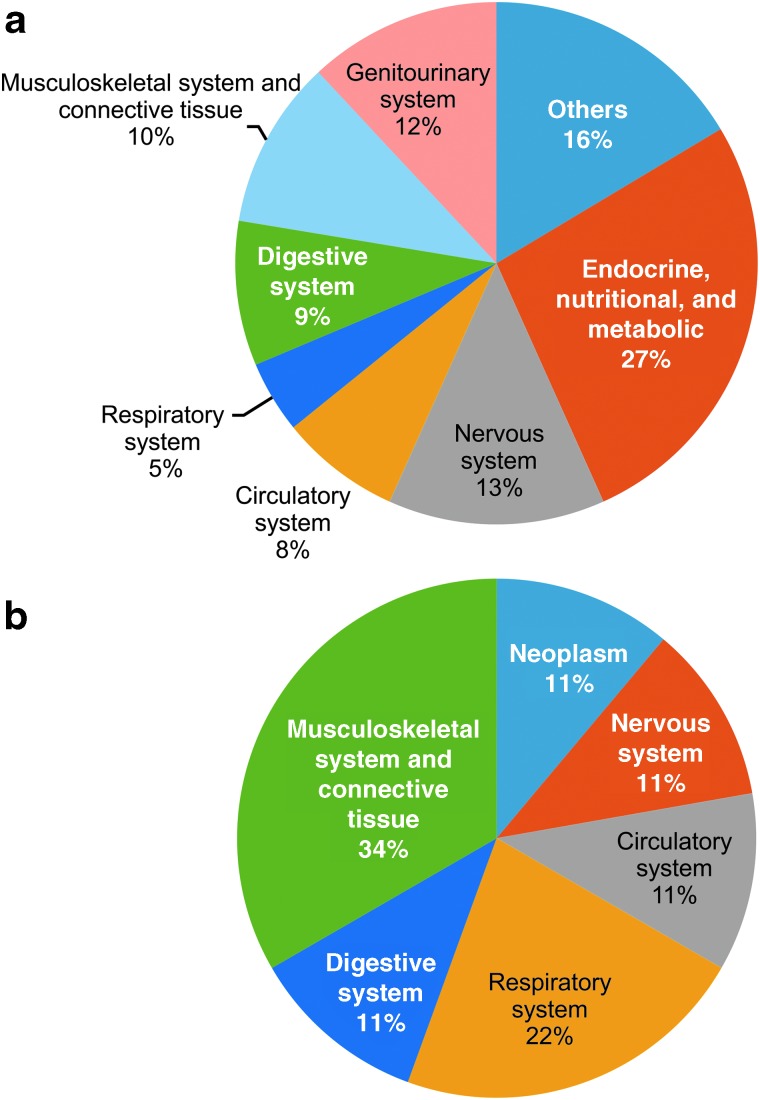
Reasons for referral to local clinics **(a)** and hospitals **(b)**. Color images are available online.

The most common diagnostic category for cases referred to local clinics by the consulting doctor was also endocrine, nutritional, and metabolic diseases (27%) followed by disease of the nervous system (13%) and the genitourinary system (12%) (*Fig. 3a*). For cases referred to a tertiary hospital, the most common diagnostic category was diseases of the musculoskeletal system and connective tissue (34%) followed by those of the respiratory system (22%) (*Fig. 3b*).

## Discussion

Research into the actual experiences of telehealth counseling program for expatriates has been rare. To the best of our knowledge, this study was the first to conduct the proof-of-concept test for providing medical counseling to expatriates and showed the feasibility, as well as acceptability of a telehealth counseling program.

Expatriates are not well studied, but they are a distinct population exposed to the risk of health problems related to travel exposure.^10^ A recent report by the Finaccord company^11^ found that there are roughly 50.5 million expatriates worldwide, and this number is expected to grow over the next few years. An effective healthcare system for this group is needed, and one solution might be healthcare services provided through the telehealth system. Although our telehealth counseling system cannot respond to emergent or urgent situations or prescribe adequate treatments, this system played a role in fulfilling expatriates' unmet needs.

Actually our study design is nothing particular compared with other telemedicine clinics in many countries. Except that the centers are located in three different countries in three different time zones, doctors are working in another country and there have been lots of network connectivity issues, privacy and confidentiality issues, prescription issues, and legal, regulatory, and economic issues.

Rapid globalization has produced a growing demand for healthcare management for expatriates or immigrants all over the world. But the capability of already existing health systems sometimes cannot adapt to the healthcare needs of these expanding vulnerable populations.

There have also been many technical, clinical, privacy, regulatory, legal, and economic issues when tele-counseling occurs in a cross-border context. Our system could be better in fulfilling the healthcare needs of these populations in a more convenient, yet safe and reliable, way. However, in this study, we have started in a small way to investigate the management of expatriates through telehealth counseling and showed its potential as one of the effective solutions.

Except for urgent or emergent cases, around two thirds of expatriates' healthcare needs could be fulfilled solely through one-time telehealth counseling. Patients can see that their symptoms are warning signs or they are just transient minor problems that can be spontaneously resolved with a doctor's advice or comments. In this regard, telehealth counseling program can keep patients from unnecessary medical service utilization and might save patients time and expense. Most of their symptoms pertained to endocrine, nutritional, and metabolic diseases or circulatory system ailments such as high blood pressure or diabetes. Tele-counseling for healthy behaviors to control high blood pressure or elevated serum glucose might have potential in chronic disease management among expatriates. Another study of this project showed a significant decrease in systolic blood pressure among expatriates with high blood pressure with our telehealth counseling program.^9^ Thus our program might have the potential to solve unmet healthcare issues in expatriates despite many existing limitations.

Cases (80, 18.1%) that needed to be seen by primary doctors at the local clinic were also mostly for simple laboratory tests to diagnose diabetes or thyroid disease. Cases (17, 3.8%) that needed to be treated at tertiary hospital or seen by a specialist were for severe pains in the back or joints. In all three countries, patients easily accessed medicines at pharmacies without a doctor's prescription, and sometimes inappropriate medicines, incorrect information, and lack of medical knowledge worsened their health status. Hence, further advancements in telehealth counseling systems will be needed for accurate diagnosis and to develop closer connections with local hospitals that have diagnostic equipment or management tools to ensure effective referrals.

Legal issues can be especially challenging in our telemedicine system for expatriates. The framework of cross-border telemedicine consists of both international and national law. According to the telemedicine service guideline by the Korea Health Industry Development Institute,^12^ doctor-to-doctor and doctor-to-nurse services for patients and cooperative services among medical professionals of the community health centers or hospitals in the medically underserved areas are allowed, but a direct doctor-to-patient service is considered illegal. As far as we know, there is no common legal framework in international telemedicine services, and relevant national regulations did not exist in the three countries where we provided services. But in general, documented consent from patients, as well as healthcare providers, should be obtained, the physical environment of telemedicine rooms should be private with no external noise and disturbance, education before telemedicine encounters should be carried out (such as structures and timing of services), and specific attention should be paid to issues such as record keeping, scheduling, privacy and security, potential risks, confidentiality, mandatory reporting, billing, and any information specific to the nature of videoconferencing. There are also clinical perspectives, technical perspectives, and privacy issues in several guidelines regarding the implementation of telemedicine.^13,14^

The lawyer consulting on our project documented that it was legally performed because the system allowed for doctor-to-nurse communication, and expatriates living in resource-limited countries can be regarded as a medically underserved population. Doctors were allowed to check patients' history with symptoms and suggest possible diagnosis with management or second opinions with the help of coordinators, and make a referral to another hospital or emergency department in some cases, but they were not allowed to prescribe or modify a patient's medication because of legal issues. Despite these limitations, people were very satisfied with the teleconsultation, and the problems of two thirds of them were easily solved with just a single visit.

According to a suggested framework for telehealth programs in six domains,^15^ our program could be evaluated as follows. For health services, we focused on diagnosis, management, or consultation for a second opinion through real-time video communication between doctor and patient assisted by a coordinator. Moreover, our telehealth network has been connected through a VPN system with a high-speed broadband network and also through a different mode of communication for matters such as medical images or health records. From a socioeconomic viewpoint, our system costs a lot due to the high-speed secured network with center-focused and coordinator-assisted systems and does not allow much flexibility in responding to patients' healthcare needs. However, the benefits of this system are that it could result in a high-quality telehealth counseling system, as well as ensure privacy and the security of personal information.

Barriers to the use of our system included limited counseling time and the need for patients to visit the DHC for telehealth counseling service, leading to additional time and space barriers that the telemedicine had tried to overcome in the first place. This system did not permit the prescribing of medication and was limited solely to telehealth counseling due to regulatory or legal problems in connecting doctors with patients through a global network.

Despite several limitations, our telehealth counseling program proved feasible in three different countries with different telecommunication environments. The very high satisfaction rates indicated that this system helped people living with limited healthcare resources in some way. This might be due to two factors as follows: one factor was the removal of language barriers in communicating with doctors. Clear communication between healthcare providers and patients is essential because it impacts patient satisfaction, adherence to recommendations, and health outcomes,^20^ and these are influenced by language differences.^16,17^ Another factor might be a patient's trust in the doctor. In the doctor–patient relationship, trust is considered to be an essential factor, and patients with higher trust experience greater satisfaction and better health outcomes.^18^ Besides, there is an association between patients' trust in doctors and doctors' institutions.^19^ Seoul National University Bundang hospital has been among the top six tertiary hospitals in Korea for a long time, and this value could cause patients to trust the doctor's recommendations or explanations and lead to a high satisfaction rate.

To implement and sustain a telehealth system in developing countries, several requirements must be considered.^20,21^ Our goal was to deliver qualitative healthcare counseling to expatriates, but to be responsive in every situation, this system should be effectively connected with local clinics or hospitals, and transfer through a brief referral note might be beneficial. In addition, the current situation of countries should be carefully considered before implementation, because some external constraints regarding tele-networking might be applicable. Counseling fees or a business model should be considered for the sustainability of this program. Our system was funded by the government project for a year and a half, but when funds run out, sustainability issues could pose a big challenge.

Most of our DHCs were located in urban areas, and hence, chronic diseases were the main ailments reported. However, if the DHC was located in a rural area, delivering primary care service, as well as responding to emergency situations, should be the first priority.

Finally, the infrastructure of the system, such as the price of internet usage, electricity consumption rates, poor-quality telephone services, and poor supervision of healthcare staff, could be one of the biggest barriers to implement an effective telehealth system. Furthermore, lack of transportation, effective referrals, and effective medical supplies could be another barrier in sustainably managing patients through a telehealth system.^22^

Further customization in identification and assessment of healthcare needs for long-term occupational travelers or expatriates, as well as checking of infrastructure systems and provision of continual assistance, training, and guidance, will be needed.

## Conclusions

Telehealth counseling programs for expatriates were found to be feasible in several different settings in three different countries and showed high user-satisfaction rates. This system has great potential to manage people effectively without unnecessary healthcare visits. However, further efforts will be needed to implement and sustain it as an effective system.
